# Accuracy of the Safer Dx Instrument to Identify Diagnostic Errors in Primary Care

**DOI:** 10.1007/s11606-016-3601-x

**Published:** 2016-02-22

**Authors:** Aymer Al-Mutairi, Ashley N. D. Meyer, Eric J. Thomas, Jason M. Etchegaray, Kevin M. Roy, Maria Caridad Davalos, Shazia Sheikh, Hardeep Singh

**Affiliations:** Houston Veterans Affairs Center for Innovations in Quality, Effectiveness and Safety, Michael E. DeBakey Veterans Affairs Medical Center and Baylor College of Medicine, 2002 Holcombe Boulevard 152, Houston, TX 77030 USA; Department of Family & Community Medicine, Baylor College of Medicine, Houston, TX USA; Department of Internal Medicine, University of Texas Medical School at Houston, Houston, TX USA; The University of Texas at Houston-Memorial Hermann Center for Healthcare Quality and Safety, Houston, TX USA; RAND Corporation, Santa Monica, CA USA; Department of Pediatrics, Section of Critical Care Medicine, Baylor College of Medicine and Texas Children’s Hospital, Houston, TX USA; Department of Medicine, Baylor College of Medicine and Ben Taub Hospital - Harris Health System, Houston, TX USA

**Keywords:** diagnostic error, measurement, patient safety, diagnostic safety, primary care, quality improvement

## Abstract

**IMPORTANCE:**

Diagnostic errors are common and harmful, but difficult to define and measure. Measurement of diagnostic errors often depends on retrospective medical record reviews, frequently resulting in reviewer disagreement.

**OBJECTIVES:**

We aimed to test the accuracy of an instrument to help detect presence or absence of diagnostic error through record reviews.

**DESIGN:**

We gathered questions from several previously used instruments for diagnostic error measurement, then developed and refined our instrument. We tested the accuracy of the instrument against a sample of patient records (*n* = 389), with and without previously identified diagnostic errors (*n* = 129 and *n* = 260, respectively).

**RESULTS:**

The final version of our instrument (titled Safer Dx Instrument) consisted of 11 questions assessing diagnostic processes in the patient–provider encounter and a main outcome question to determine diagnostic error. In comparison with the previous sample, the instrument yielded an overall accuracy of 84 %, sensitivity of 71 %, specificity of 90 %, negative predictive value of 86 %, and positive predictive value of 78 %. All 11 items correlated significantly with the instrument’s error outcome question (all *p* values ≤ 0.01). Using factor analysis, the 11 questions clustered into two domains with high internal consistency (initial diagnostic assessment, and performance and interpretation of diagnostic tests) and a patient factor domain with low internal consistency (Cronbach’s alpha coefficients 0.93, 0.92, and 0.38, respectively).

**CONCLUSIONS:**

The Safer Dx Instrument helps quantify the likelihood of diagnostic error in primary care visits, achieving a high degree of accuracy for measuring their presence or absence. This instrument could be useful to identify high-risk cases for further study and quality improvement.

**Electronic supplementary material:**

The online version of this article (doi:10.1007/s11606-016-3601-x) contains supplementary material, which is available to authorized users.

## INTRODUCTION

Despite the success of hospital-based patient safety efforts, progress to improve the safety of primary care has lagged.^1^–^4^ A recent Institute of Medicine (IOM) report “Improving Diagnosis in Health Care”^5^ highlights the safety implications of diagnostic errors, which are one of the most common types of medical errors in primary care.^6^–^13^ These errors are estimated to affect about one in 20 US adults in outpatient settings annually^14^ and are the leading basis for ambulatory malpractice claims.^7^,^15^ Diagnostic errors have remained under-studied in patient safety research,^12^,^16^ partly because they are difficult to measure.^17^–^20^ Measurement of diagnostic errors often depends heavily on detailed retrospective review of patients’ medical records. Clinicians do not always agree on the presence or absence of error, and details about the clinical situation are often absent when making judgments in hindsight.^21^,^22^ Additionally, diagnoses often require additional testing or consultations for confirmation and evolve over time.^23^ Not surprisingly, studies consistently demonstrate low inter-physician agreement, or accuracy, on medical record reviews for diagnostic errors.^24^–^30^

National initiatives such as maintenance of certification and physician quality reporting systems have placed an increasing emphasis on ambulatory quality and safety. The IOM report on improving diagnosis^5^ also recommends a comprehensive and rigorous methodology to measure diagnostic errors to advance the science in this area and reduce their burden.^22^,^31^–^34^ In our previous work, we used judgments from multiple physician-raters to determine diagnostic error in selected primary care visit-records.^9^,^35^–^37^ We defined diagnostic errors as missed opportunities to make a correct or timely diagnosis based on the available evidence, regardless of patient harm.^35^ We considered diagnostic errors to have occurred when at least two independent physician reviewers confirmed their presence. While reviewers used a structured data collection instrument to help them evaluate the records, they relied on subjective assessments to make judgments. Despite extensive training and calibration efforts, the reviewers only reached fair agreement.^36^ To facilitate better measurement through medical record reviews, we developed a new structured instrument consisting of objective criteria to improve the accuracy of assessing diagnostic errors.

## METHODS

### Study Design

After institutional board review approval, we gathered questions from several previously used instruments for diagnostic error measurement^10^,^16^,^35^ and used an operational definition of diagnostic error^37^ to develop an initial draft of the instrument. We iteratively refined our instrument through pilot medical record reviews and multidisciplinary input, and tested the accuracy of the final instrument by conducting reviews of a sample of patients with and without diagnostic errors.

### Study Setting

The study site was a large urban VA facility with 35 full-time primary care providers (PCPs), including physicians, physician assistants, and nurse practitioners, providing comprehensive care to approximately 50,000 patients. It had an integrated and well-established electronic health record (EHR), and large clinic networks through which it provided longitudinal care to ethnically and socioeconomically diverse patients from rural and urban areas. Most PCPs were physicians, some of whom supervised residents, and visits included scheduled follow-up visits and “drop-in” unscheduled visits.

### Instrument Development

We developed a 12-item rating instrument (the Safer Dx Instrument) for the purpose of determining the presence or absence of diagnostic error for a specific episode of care. Our team consisted of five practicing clinicians (three of who were also diagnostic error and/or quality improvement experts), a psychometrician and a cognitive psychologist. We first sought existing content from instruments previously used in research on diagnostic error measurement.^10^,^16^,^35^ We then adapted some items from these previous instruments and added additional items to address important aspects of the diagnostic process such as history-taking, physical examination, test ordering, and test interpretation. All of the questions were intended to identify missed opportunities in diagnosis using criteria developed through several previous studies.^9^,^35^,^36^ We relied heavily on three clinical criteria found to be useful in our previous work to determine the presence or absence of diagnostic errors, i.e., case analysis reveals evidence of missed opportunity to make a correct or timely diagnosis; missed opportunity was framed within the context of an “evolving” diagnostic process; and opportunity could be missed by the provider, care team, system, and/or patient (see online Supplementary Appendix for details on criteria and instrument development).^37^

The final version of the Safer Dx Instrument consisted of 11 questions regarding the appropriateness of the diagnostic process and one summary question regarding the overall impression of diagnostic error (Table 1). Items were scored from 1 (strongly agree an error occurred) to 6 (strongly disagree that an error occurred), with the exception of three items (items 6, 9, and 10) that were reverse scored. Items were rated on a six-point Likert scale in order to allow for “gray areas” in the determination of diagnostic error (i.e., we did not want to force someone to say “absolutely an error” vs. “absolutely not an error,” but instead select response options that were less definite). However, to directly compare the overall impression of diagnostic error in item 12 to a previous sample of patients with and without diagnostic errors, item 12 (the main outcome) was dichotomized, such that 1 to 3 represented diagnostic error and 4 to 6 represented absence of diagnostic error (alternate ways to dichotomize are included in the online Appendix Table).

**Table 1.** The Safer Dx Instrument: Items for Determining Presence or Absence of Diagnostic Error in a Primary Care Encounter

Rate the following items for the episode of care under review^a^:	
1---2---3---4---5---6	
1 = Strongly Agree	6 = Strongly Disagree
1. The history that was documented at the patient–provider encounter was suggestive of an alternate diagnosis, which was not considered in the assessment.	
2. The physical exam documented at the patient–provider encounter was suggestive of an alternate diagnosis, which was not considered in the assessment.	
3. Diagnostic testing data (laboratory, radiology, pathology or other results) associated with the patient–provider encounter were suggestive of an alternate diagnosis, which was not considered in the initial assessment.	
4. The diagnostic process at the initial assessment was affected by incomplete or incorrect clinical information given to the care team by the patient or their primary caregiver.	
5. The clinical information (i.e., history, physical exam or diagnostic data) present at the initial assessment should have prompted additional diagnostic evaluation through tests or consults.	
6. The initial assessment at an earlier visit was appropriate, given the patient’s medical history and clinical presentation.	
7. Alarm symptoms or “Red Flags” (i.e., features in the clinical presentation that are considered to predict serious disease) were not acted upon at an earlier assessment.	
8. Diagnostic data (laboratory, radiology, pathology or other results) available or documented at the initial assessment were misinterpreted in relation to the subsequent final diagnosis.	
9. The differential diagnosis documented at the initial assessment included the subsequent final diagnosis.	
10. The final diagnosis was an evolution of the initial presumed diagnosis.	
11. The clinical presentation was not typical of the final diagnosis.	
12. In conclusion, based on all the above questions, the episode of care under review had a diagnostic error.	

^a^In all questions, a rating of 1 most likely represented a diagnostic error and a rating of 6 indicated that no error was identified, except questions 6, 9 and 10 where ratings were reversed

Two physicians on our multidisciplinary team (AA and CD) pilot tested the instrument and provided feedback, which was used in team meetings for further refinement. The instrument was further refined through an iterative process of reviews by five additional practicing physicians outside of this team to ensure content and face validity. This type of approach is consistent with standard survey item development practices.^38^ Details on pilot testing are provided in the online Appendix. The chart reviewer, an actively practicing board-certified primary care physician (AA) with experience in EHR and patient safety projects, was trained extensively on record reviews.

#### Sample/Participants

We tested the Safer Dx Instrument using a cohort of 389 patients with and without diagnostic errors (*n* = 129 and *n* = 260, respectively) from the VA site in our prior study.^35^ At this VA study site, 1300 records had been selected for review; 886 using a “trigger” algorithm to identify patients with possible diagnostic errors based on unexpected hospitalizations and return visits, and 414 as “trigger negative” controls. After exclusion of false positives with no or minimal information available for error assessment, 1169 records remained and were reviewed in detail by at least two independent raters to determine the presence or absence of diagnostic errors. Patients were mostly male (93.8 %); 56.8 % White and 39 % Black. The cases represented a heterogeneous group of common medical conditions seen in the primary care setting and were independent of cases used to develop and pilot-test the earlier draft of the instrument.

#### Outcomes

The physician-reviewer blinded to the diagnostic error outcome reviewed medical records from all 389 patients and completed the Safer Dx Instrument for each. Clinical details were determined through detailed reviews of the EHR about care processes at an index primary care visit and subsequent visits. The reviewer evaluated EHR data up to 1 year after the index visit to help determine the clinical context. A second reviewer (board certified in internal medicine, but otherwise with similar familiarity with EHRs) independently assessed a random sample of 30 records from the testing data set (ten with and 20 without errors).

#### Statistical Analysis

We calculated the Safer Dx Instrument’s overall sensitivity, specificity, positive predictive value, and negative predictive value by comparing the main, dichotomized outcome from item 12 (1–3 = error, 4–6 = no error as determined by the single physician using the instrument) to results obtained in the previous study.^35^ Accuracy was defined as physician agreement with presence or absence of diagnostic errors as compared to our previous study results for all 389 cases.^35^

Additionally, we examined whether any of the 11 diagnostic process items were related to the main outcome (i.e., the rater’s overall impression of diagnostic error) by computing both Spearman correlation coefficients (using the six-point scaled outcome) and Pearson correlations coefficients (using the dichotomized outcome). All items that were significantly correlated to the main outcome were entered into a factor analysis with varimax rotation to identify any higher-order dimensions represented by clusters of items. We kept dimensions with eigenvalues over Kaiser’s criterion of 1 and assessed the internal consistency of the resulting dimensions using Cronbach’s alpha.

Finally, we developed a score based on all of the instrument items to predict whether cases assessed via Safer Dx Instrument were determined to be errors in our previous study. We thus performed a logistic regression using summed scores from the dimensions obtained in the factor analysis above, as well as individual items not included in the dimensions, to predict whether each case was an error or not. Using the obtained regression equation, we compared scores obtained in the error cases and the non-error cases. This would allow users to create potential cut-off scores, signaling lower or higher likelihood of diagnostic error. Users would have the flexibility to personalize these cutoff scores depending on how inclusive and conservative they wanted to be.

## RESULTS

Of 389 patient records, use of the instrument identified 117 as diagnostic errors as compared to 129 from our previous sample. The dichotomized score on Safer Dx Instrument’s main outcome of interest (presence or absence of diagnostic errors, i.e., 1–3 = error, 4–6 = no error), was associated with an overall accuracy of 84 %, sensitivity of 71 %, specificity of 90 %, negative predictive value of 86 %, and positive predictive value of 78 % for detecting diagnostic errors. Alternate splits of the six-point scale can be seen in the online Appendix Table.

Items 1–11 were all significantly correlated with item 12 (global impression of diagnostic error; see Spearman and Pearson correlation analyses, Table 2). The Kaiser-Meyer-Olkin measure verified the sampling adequacy for the factor analysis, KMO = 0.87. Three dimensions had eigenvalues over Kaiser’s criterion of 1 and in combination explained over 76 % of the variance. As such, three domains were kept. The first domain (initial diagnostic assessment) included questions 1, 2, 5–7, 9, and 10; the second domain (performance and interpretation of diagnostic tests) included questions 3 and 8; and the third domain (patient factors) included questions 4 and 11. Cronbach’s alpha coefficients associated with these groups were 0.93, 0.92, and 0.38, respectively, suggesting that the first and second domains have an excellent internal consistency and reliability, while the third domain showed poor internal consistency.

**Table 2.** Correlations Between the 11 Diagnostic Process Instrument Items and the Safer Dx Instrument Outcome (Diagnostic Error vs. No Error) in 389 Cases

The Safer Dx Instrument items	*Spearman’s correlation between item and error outcome rho (*p* value)	*Pearson correlation between item and error outcome r (*p* value)
1. The history that was documented at the patient–provider encounter was suggestive of an alternate diagnosis, which was not considered in the assessment.	0.67 (< 0.001)	0.61 (< 0.001)
2. The physical exam documented at the patient–provider encounter was suggestive of an alternate diagnosis, which was not considered in the assessment.	0.50 (< 0.001)	0.44 (< 0.001)
3. Diagnostic testing data (laboratory, radiology, pathology or other results) associated with the patient–provider encounter were suggestive of an alternate diagnosis, which was not considered in the assessment.	0.47 (< 0.001)	0.48 (< 0.001)
4. The diagnostic process at the initial assessment was affected by incomplete or incorrect clinical information given to the care team by the patient or primary caregiver.	0.17 (.001)	0.15 (0.004)
5. The clinical information (i.e., history, physical exam and diagnostic data) present at the initial assessment should have prompted additional diagnostic evaluation through tests or consults.	0.75 (< 0.001)	0.72 (< 0.001)
**6. The initial assessment at an earlier visit was appropriate given the patient’s medical history and clinical presentation.	0.81 (< 0.001)	0.75 (< 0.001)
7. Alarm symptoms or “Red Flags” (i.e., features in the clinical presentation that are considered to predict serious disease) were not acted upon at an earlier assessment.	0.74 (< 0.001)	0.75 (< 0.001)
8. Diagnostic data (laboratory, radiology, pathology or other results) available or documented at the initial assessment were misinterpreted in relation to the subsequent final diagnosis.	0.45 (< 0.001)	0.47 (< 0.001)
**9. The differential diagnosis documented at the initial assessment included the subsequent final diagnosis.	0.80 (< 0.001)	0.79 (< 0.001)
**10. The final diagnosis was an evolution of the initial presumed diagnosis.	0.75 (< 0.001)	0.72 (< 0.001)
11. The clinical presentation was not typical given the final diagnosis.	0.48 (< 0.001)	0.48 (< 0.001)

* Spearman’s correlation uses the main outcome as a six-item scale, whereas the Pearson’s correlations use the main outcome dichotomy, such that 1–3 are considered errors and 4–6 are considered non-errors

** These items were reverse scored

**Figure 1.**
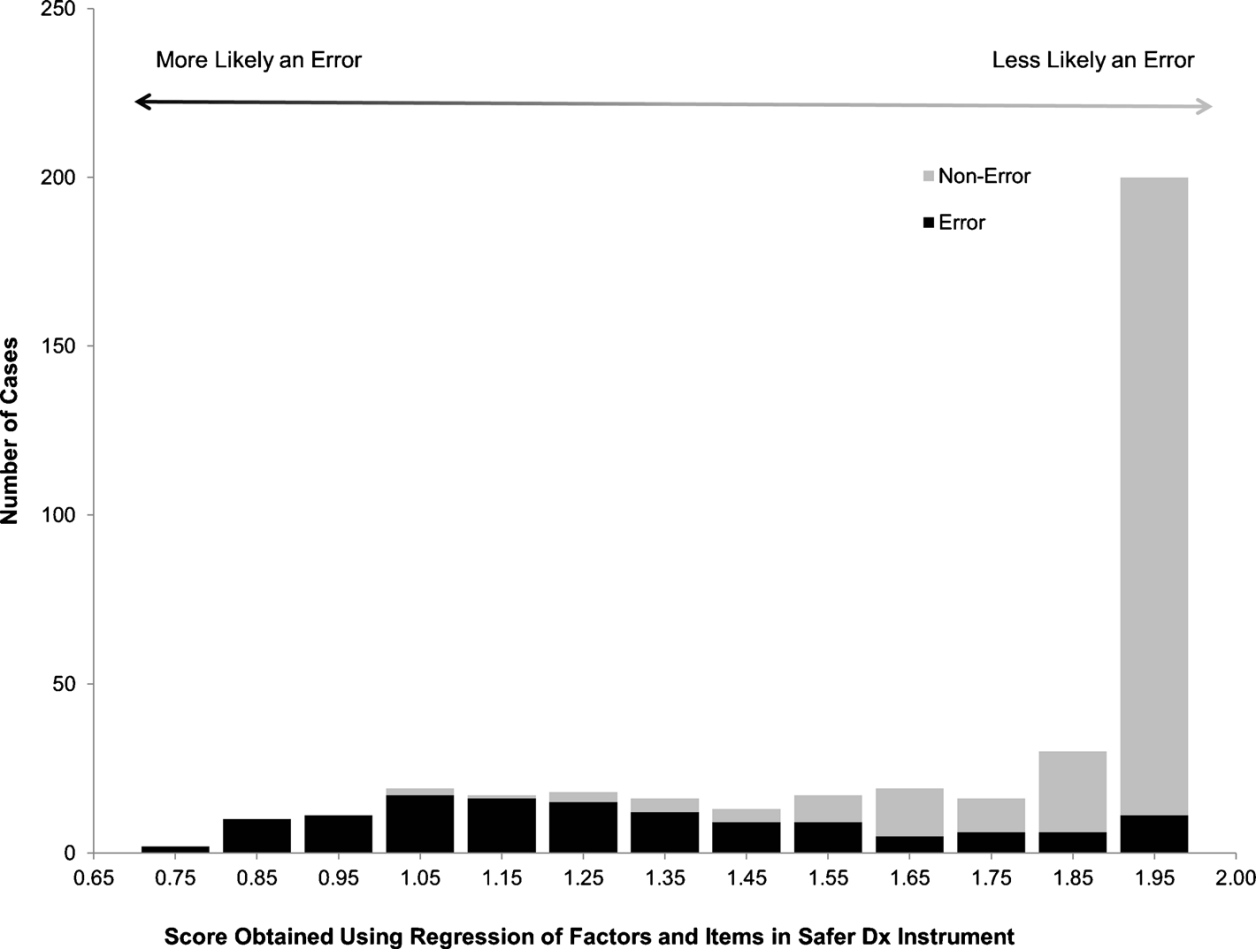
Relationship between diagnostic error status and scores obtained using the safer Dx instrument scoring system.

**Figure 2.**
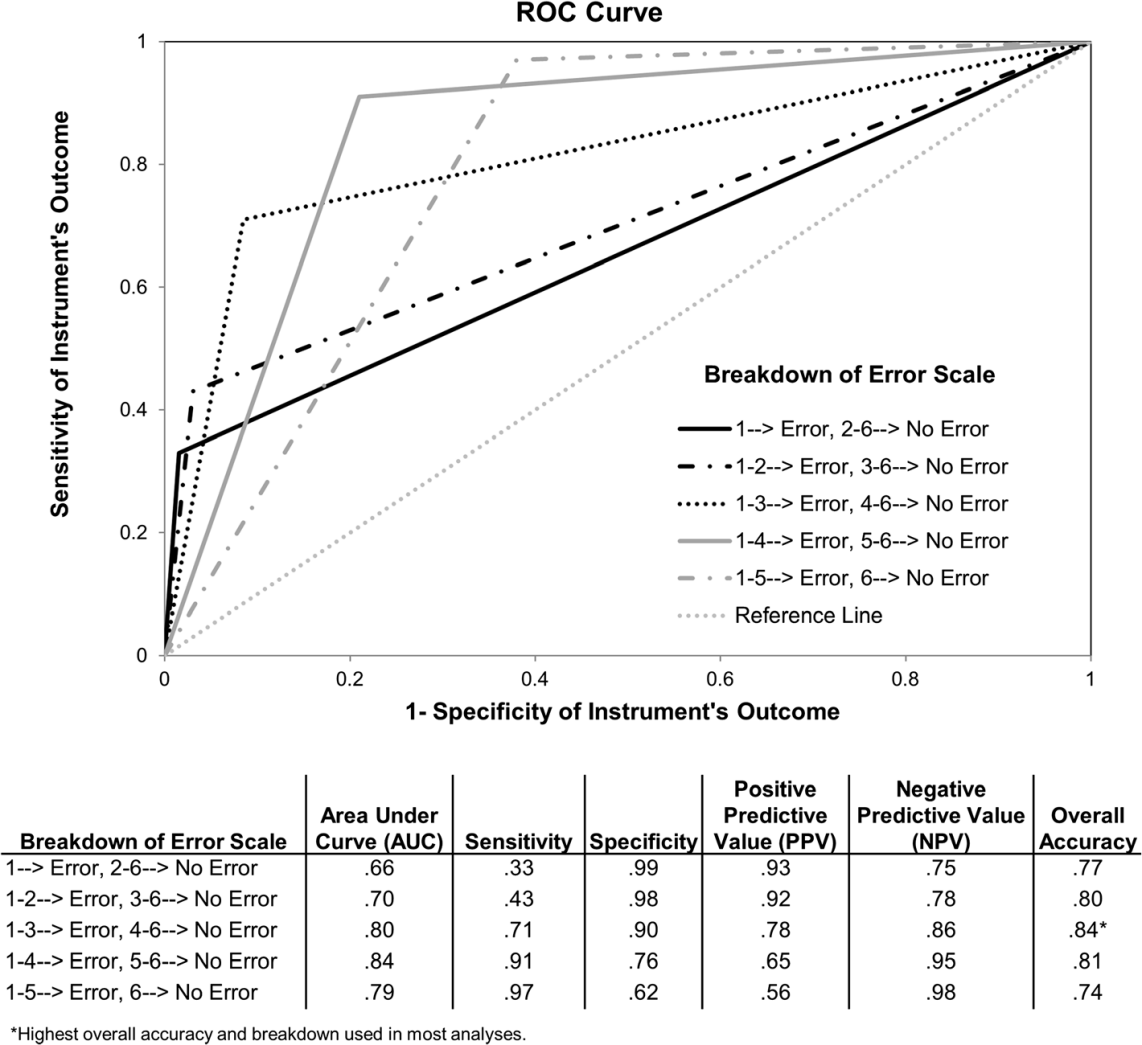
ROC curve for safer Dx instrument’s characteristics.

To create an overall score for the instrument that could predict the likelihood that a reviewed case involved a diagnostic error or not, we summed scores from each item within a dimension to create factor scores. However, because of the poor internal consistency of the third domain (questions 4 and 11), we retained these two items as individual items and did not conceive them as forming a specific factor to create the scoring system. Factor scores and items 4 and 11 were then entered into a multivariate logistic regression with error versus no error as the predicted outcome (as determined from the previous study). The summed factors and two individual items significantly predicted presence of diagnostic error in the previous study: *F*(4 383) = 117, *p* < 0.001, R^2^ = 0.55. Using the obtained formula, where *Error Score* = 0.395 + (Σ_Factor1Items_*0.03) + (Σ_Factor2Items_*0.003) + (Item 4 * −0.005) + (item 11 * 0.05), we created a figure showing the frequency of different scores in error versus no error cases. As shown in Fig. 1, lower scores are more associated with errors and higher scores are less associated with errors. Cutoff scores can be created to distinguish between diagnostic error and non-error cases and can also be used to create different risk groups; such as high, moderate, and low risk of diagnostic error. These cutoff scores could be personalized depending on a user’s desire to trade-off between positive predictive and negative predictive value, as well as between sensitivity and specificity. For example, in the future, a practice or an institution might decide to use a cutoff score of ≤ 1.50 to indicate the presence of diagnostic error and a score of ≥ 1.90 to indicate its absence. The advantage of using scoring systems such as this one is that practices or institutions might be able use scores to categorize patients into high risk, moderate risk, and low risk for diagnostic errors in order to flag cases in need of further review and analysis. An ROC curve for Safer Dx Instrument’s performance characteristics is shown in Fig. 2.

The second independent review on the randomly selected 30 patients revealed the following: agreement with previous study sample =73.3 %; agreement with current sample = 83.3 % and agreement with either previous study sample or current sample =86.7 %.

## DISCUSSION

Novel approaches are needed to address the challenges of measuring diagnostic error in primary care settings.^17^ In response to this need, we developed the Safer Dx Instrument to measure diagnostic errors and tested its accuracy to help detect their presence or absence via record reviews. Using a sample of previously confirmed cases, we found that the Safer Dx Instrument had a reasonably high accuracy and predictive value to detect presence or absence of diagnostic error. The Safer Dx Instrument is a first step in standardizing the measurement of diagnostic processes in the primary care setting through record review and could help providers and/or healthcare facilities detect potential diagnostic errors for further review using a single reviewer. The instrument’s items clustered into two important diagnostic process domains with face validity (initial diagnostic assessment and performance and interpretation of diagnostic tests). A third, potentially important domain (patient factors) was discovered but had poor internal consistency; therefore, future work should explore developing additional items to measure patient factors.

Without measuring diagnostic performance, we are largely in the dark about an important task performed by primary care physicians.^39^ There are no standardized tools or strategies to facilitate measurement of diagnostic performance in the complex and vulnerable primary care setting. The Safer Dx Instrument can be used to guide a comprehensive assessment of the patient’s diagnostic experience through a detailed examination of all aspects of the patient’s medical record, including patient history, physician examination, interpretation of diagnostic tests, ordering of additional testing or referrals, generating a differential diagnosis and initial medical assessment, and evaluating the initial diagnosis or related complications. Therefore, the instrument’s 11 items address a wide spectrum of diagnostic process breakdowns that have been described in primary care.^10^,^16^

The Safer Dx Instrument would likely be most effective when used in combination with trigger algorithms to select a “high-risk” cohort of medical records^36^ to review versus reviewing random or non-selected records. A trigger and review strategy could provide an effective screen for diagnostic errors in primary care settings, and could be followed by a secondary review of selected records by one or more physicians to confirm errors and/or to initiate further analysis. Currently, there are no such methods being used in primary care. Although this technique cannot identify all errors, it will be a useful start to enhance learning and feedback about diagnostic safety in primary care settings. Because of reduced reliance on subjectivity, this instrument could also improve agreement on diagnostic errors.

In addition to being used retrospectively to identify cases at highest need for secondary review, the instrument could be used for learning and feedback on what aspects of the diagnostic process broke down. This exercise could lead to a more intensive analysis of diagnoses at a practice level and raise awareness of diagnostic safety issues in the primary care setting. As the recent IOM report also notes,^5^ measurement of diagnostic errors is essential to create the necessary policy and practice initiatives to improve safety in this area.^40^

Our study has several limitations. We focused solely on primary care patients and relied on an integrated and comprehensive EHR review to evaluate clinical details about visits, tests, procedures, and referrals. These details might not be available in other primary care practices that are not integrated with other health care settings. However, this is likely to change over time, as several national initiatives are addressing improved integration and data exchange for primary care records. We used an existing data set and a specific trigger algorithm to identify most cases, which may have contributed to a selection bias toward patients with return-visits who might be at more risk for error. However, as there are no currently available practical methods to find diagnostic errors in primary care, any new tools first need rigorous testing. Error determination was dependent on accurate record-keeping and could be confounded by documentation related limitations and hindsight bias.^30^,^41^ Measuring an evolving diagnostic process fraught with uncertainty is challenging.^23^ Individual reviewers would also vary in their tolerance of ambiguity and their perspectives regarding utilization of diagnostic testing. The use of the instrument involves some amount of individual judgment, even though we tried to minimize this. However, the instrument guides a reviewer through most concepts that need to be considered while analyzing the diagnostic process for problems within a clinical encounter. Moreover, our strategy of a single clinician who can effectively screen records for a subsequent detailed review by an additional team of clinicians would likely be more feasible and acceptable to others. We also acknowledge that agreement between our two reviewers was not perfect, but believe it is a start for measuring something so important but yet quite abstract (this concept is also acknowledged in the recent IOM report). The instrument might perform differently in different populations and different disease conditions and thus, testing will be required in other settings. Additional scientific understanding in the future will likely make this instrument better.

In conclusion, we tested a new instrument and found it to have a high degree of accuracy and predictive value for measuring diagnostic errors in primary care settings. This instrument could be useful to identify high-risk cases for further study and quality improvement. With further testing in additional clinical settings, the Safer Dx Instrument could be used to enhance knowledge on improving diagnostic safety in primary care settings.

## Electronic supplementary material

Below is the link to the electronic supplementary material.

ESM 1 (DOCX 30 kb)

## References

[1] Efforts To Improve Patient Safety Result in 1.3 Million Fewer Patient Harms. Interim Update on 2013 Annual Hospital-Acquired Condition Rate and Estimates of Cost Savings and Deaths Averted From 2010 to 2013 2014 Dec ; Publication # 15-0011-EF Available at: http://www.ahrq.gov/professionals/quality-patient-safety/pfp/interimhacrate2013.html. Accessed Jan 4 2016.

[2] Ely JW, Kaldjian LC, D’Alessandro DM (2012). Diagnostic errors in primary care: lessons learned. J Am Board Fam Pract.

[3] Schiff GD, Puopolo AL, Huben-Kearney A, Yu W, Keohane C, McDonough P (2013). Primary care closed claims experience of Massachusetts malpractice insurers. JAMA Intern Med.

[4] Institute of Medicine (IOM). Engineering a Learning Healthcare System: A Look at the Future: Workshop Summary 2011 Available at: http://www.nap.edu/openbook.php?record_id=12213&page=R2. Accessed Jan 4 2016. 21977540

[5] National Academies of Sciences Engineering and Medicine (2015). Improving diagnosis in health care.

[6] Bishop TF, Ryan AM, Casalino LP (2011). Paid malpractice claims for adverse events in inpatient and outpatient settings. JAMA.

[7] Gandhi TK, Kachalia A, Thomas EJ, Puopolo AL, Yoon C, Brennan TA (2006). Missed and delayed diagnoses in the ambulatory setting: a study of closed malpractice claims. Ann Intern Med.

[8] Phillips RL, Bartholomew LA, Dovey SM, Fryer GE, Miyoshi TJ, Green LA (2004). Learning from malpractice claims about negligent, adverse events in primary care in the United States. Qual Saf Health Care.

[9] Singh H, Thomas EJ, Khan MM, Petersen LA (2007). Identifying diagnostic errors in primary care using an electronic screening algorithm. Arch Intern Med.

[10] Singh H, Weingart SN (2009). Diagnostic errors in ambulatory care: dimensions and preventive strategies. Adv Health Sci Educ Theory Pract.

[11] Singh H, Graber M (2010). Reducing diagnostic error through medical home-based primary care reform. JAMA.

[12] Newman-Toker DE, Pronovost PJ (2009). Diagnostic errors--the next frontier for patient safety. JAMA.

[13] **Singh H, Graber ML.** Improving diagnosis in health care - the next imperative for patient safety. N Engl J Med. 2015. 10.1056/NEJMp1512241 26559457

[14] Singh H, Meyer AN, Thomas EJ (2014). The frequency of diagnostic errors in outpatient care: estimations from three large observational studies involving US adult populations. BMJ Qual Saf.

[15] Schiff GD, Hasan O, Kim S, Abrams R, Cosby K, Lambert BL (2009). Diagnostic error in medicine: analysis of 583 physician-reported errors. Arch Intern Med.

[16] **Schiff GD, Kim S, Abrams R, Cosby K, Lambert B, Elstein AS**. Diagnosing diagnostic errors: Lessons from a multi-institutional collaborative project. In Advances in Patient Safety: From Research to Implementation (Volume 2: Concepts and Methodology). Rockville, MD.: Agency for Healthcare Research and Quality AHRQ Publication Nos. 050021 (1–4).; 2005. p. 255–78.

[17] Wachter RM (2010). Why diagnostic errors don’t get any respect--and what can be done about them. Health Aff (Millwood).

[18] Giardina TD, King BJ, Ignaczak AP, Paull DE, Hoeksema L, Mills PD (2013). Root cause analysis reports help identify common factors in delayed diagnosis and treatment of outpatients. Health Aff (Millwood).

[19] Singh H (2013). Diagnostic errors: moving beyond ‘no respect’ and getting ready for prime time. BMJ Qual Saf.

[20] Graber ML, Trowbridge RL, Myers JS, Umscheid CA, Strull W, Kanter MH (2014). The next organizational challenge: finding and addressing diagnostic error. Jt Comm J Qual Patient Saf.

[21] Thomas EJ, Petersen LA (2003). Measuring errors and adverse events in health care. J Gen Intern Med.

[22] Neale G, Woloshynowych M (2003). Retrospective case record review: a blunt instrument that needs sharpening. Qual Saf Health Care.

[23] Zwaan L, Singh H (2015). The challenges in defining and measuring diagnostic error. Diagnosis.

[24] Localio AR, Weaver SL, Landis JR, Lawthers AG, Brenhan TA, Hebert L (1996). Identifying adverse events caused by medical care: degree of physician agreement in a retrospective chart review. Ann Intern Med.

[25] Forster AJ, Taljaard M, Bennett C, van Walraven C (2012). Reliability of the peer-review process for adverse event rating. PLoS One.

[26] Thomas EJ, Lipsitz SR, Studdert DM, Brennan TA (2002). The reliability of medical record review for estimating adverse event rates. Ann Intern Med.

[27] Brennan TA, Leape LL, Laird NM, Hebert L, Localio AR, Lawthers AG (1991). Incidence of adverse events and negligence in hospitalized patients. Results of the Harvard Medical Practice Study I. N Engl J Med.

[28] Forster AJ, O’Rourke K, Shojania KG, van Walraven C (2007). Combining ratings from multiple physician reviewers helped to overcome the uncertainty associated with adverse event classification. J Clin Epidemiol.

[29] Vincent C, Neale G, Woloshynowych M (2001). Adverse events in British hospitals: preliminary retrospective record review. BMJ.

[30] Hayward RA, Hofer TP (2001). Estimating hospital deaths due to medical errors: preventability is in the eye of the reviewer. JAMA.

[31] Graber M (2005). Diagnostic errors in medicine: a case of neglect. Jt Comm J Qual Improv.

[32] Worster A, Bledsoe RD, Cleve P, Fernandes CM, Upadhye S, Eva K (2005). Reassessing the methods of medical record review studies in emergency medicine research. Ann Emerg Med.

[33] Gilbert EH, Lowenstein SR, Koziol-McLain J, Barta DC, Steiner J (1996). Chart reviews in emergency medicine research: where are the methods?. Ann Emerg Med.

[34] Vincent C, Burnett S, Carthey J (2014). Safety measurement and monitoring in healthcare: a framework to guide clinical teams and healthcare organisations in maintaining safety. BMJ Qual Saf.

[35] Singh H, Giardina TD, Meyer AN, Forjuoh SN, Reis MD, Thomas EJ (2013). Types and origins of diagnostic errors in primary care settings. JAMA Intern Med.

[36] Singh H, Giardina TD, Forjuoh SN, Reis MD, Kosmach S, Khan MM (2012). Electronic health record-based surveillance of diagnostic errors in primary care. BMJ Qual Saf.

[37] Singh H (2014). Editorial: helping health care organizations to define diagnostic errors as missed opportunities in diagnosis. Jt Comm J Qual Patient Saf.

[38] U.S. Survey Research: Questionnaire design 2015 Available at: http://www.pewresearch.org/methodology/u-s-survey-research/questionnaire-design/. Accessed Jan 4 2016.

[39] Singh H, Sittig DF (2015). Advancing the science of measurement of diagnostic errors in healthcare: the Safer Dx framework. BMJ Qual Saf.

[40] Institute of Medicine (IOM). Activity - Diagnostic error in health care 2014 Available at: http://www.iom.edu/Activities/Quality/DiagnosticErrorHealthCare.aspx. Accessed Jan 4 2016.

[41] Caplan RA, Posner KL, Cheney FW (1991). Effect of outcome on physician judgments of appropriateness of care. JAMA.

