# Supplementary material for: Accuracy of the Safer Dx Instrument to Identify Diagnostic Errors in Primary Care
Source: J Gen Intern Med. 2016 Feb 22;31(6):602–8. doi: 10.1007/s11606-016-3601-x (PMC4870415; doi:10.1007/s11606-016-3601-x)
Supplement: Supplementary file 1 — (DOCX 30 kb) [file 11606_2016_3601_MOESM1_ESM.docx]

**Supplementary Appendix: Developmental Criteria and Pilot Testing of Safer Dx Instrument**

Developmental criteria for determining presence or absence of diagnostic errors

Development of Safer Dx Instrument was heavily informed by three criteria found to be useful in our previous work to determine the presence of diagnostic errors:^1^

*1) The case analysis reveals evidence of a missed opportunity to make a correct or timely diagnosis,* i.e., something different could have been done to make the correct diagnosis earlier.

*2) A missed opportunity is framed within the context of an “evolving” diagnostic process,* i.e., error determination accounts for the temporal or sequential context of events. Evidence of omission (failure to do the right thing) or commission (doing something wrong) exists at the particular point in time at which the “error” occurred.

*3) The opportunity could be missed by the provider, care team, system, and/or patient.* A preventable error or delay in diagnosis may occur due to factors outside the clinician’s immediate control, or when a clinician’s performance is not contributory.

For example, documentation of orthopnea, elevated venous pressures, pedal edema, chest crackles, or pulmonary edema on chest X-ray would be suggestive of heart failure as an alternative diagnosis rather than bronchitis (Questions 1-3 of the instrument) and thus suggest a missed opportunity (at an earlier index visit) to diagnose heart failure (if that indeed was found to be the subsequent final diagnosis on record review). Similarly, if a constellation of certain documented abnormal findings (e.g., cough, fever, and dyspnea) did not prompt additional evaluation with chest X-ray^2^ in a patient subsequently diagnosed with pneumonia, that would suggest a missed opportunity (Question 5). In addition, the instrument accounts for inherent uncertainty of the diagnostic process as well as the evolution of diagnosis that takes place in outpatient settings (Questions 9-11). For example, a patient with an initial diagnosis of a viral upper respiratory tract infection could present 10 days later with bacterial sinusitis, but this would not necessarily imply a missed diagnosis on the first visit. Furthermore, the clinician could have considered sinusitis in the differential diagnosis on the earlier visit but appropriately chose not to give antibiotics.

Pilot testing of the Safer Dx Instrument

We used an iterative development process to test an initial draft of the instrument using a development sample of 70 patient records (with and without diagnostic errors) from a previous study^3^ of primary care diagnostic errors in a similar patient population (mostly male, VA patients). A single board certified primary care physician (AA) from our team, blinded to the presence or absence of diagnostic error, reviewed 10-20 records at a time and discussed findings with our multidisciplinary team. The reviewer was trained extensively on record reviews, and was actively practicing in primary care and participating in projects related to patient safety and EHRs. Team discussions were used to provide feedback for further refinement of the instrument. After 50 records, changes were minimal and the reviewer conducted record reviews on an additional 20 records. In this last round, there was 75% agreement between the reviewer and presence or absence of diagnostic error as determined in the previous study.

| **Supplementary Appendix Table: PPV, NPV, Sensitivity, Specificity, and Overall Accuracy of the Safer Dx Instrument’s Error Determination Compared to Previous Study^2^** | | | | | | | | |
| --- | --- | --- | --- | --- | --- | --- | --- | --- |
|  | | | | | | | | |
| Assuming 1-5 Error; 6 No Error (Most Liberal Interpretation of Error) | | | | | | |  |  |
| PPV= | 0.56 |  |  |  |  | |  |  |
| NPV= | 0.98 |  |  |  |  | | Previous Sample | |
| Sensitivity= | 0.97 |  |  |  |  | | Error | No Error |
| Specificity= | 0.62 |  |  | Reviewer Using Safer Dx Instrument | Error | | 125 | 99 |
| Overall Accuracy= | 0.74 |  |  |  | No Error | | 4 | 161 |
|  |  |  |  |  |  | |  |  |
| Assuming 1-4 Error; 5-6 No Error | | |  |  |  | |  |  |
| PPV= | 0.65 |  |  |  |  | |  |  |
| NPV= | 0.95 |  |  |  |  | | Previous Sample | |
| Sensitivity= | 0.91 |  |  |  |  | | Error | No Error |
| Specificity= | 0.76 |  |  | Reviewer Using Safer Dx Instrument | Error | | 118 | 63 |
| Overall Accuracy= | 0.81 |  |  |  | No Error | | 11 | 197 |
|  |  |  |  |  |  | |  |  |
| **Assuming 1-3 Error; 4-6 No Error (used in analysis)** | | | | |  | |  |  |
| PPV= | 0.78 |  |  |  |  | |  |  |
| NPV= | 0.86 |  |  |  |  | | Previous Sample | |
| Sensitivity= | 0.71 |  |  |  |  | | Error | No Error |
| Specificity= | 0.90 |  |  | Reviewer Using Safer Dx Instrument | Error | | 91 | 26 |
| Overall Accuracy= | 0.84 | *Highest |  |  | No Error | | 38 | 234 |
|  |  |  |  |  |  | |  |  |
| Assuming 1-2 Error; 3-6 No Error | | |  |  |  | |  |  |
| PPV= | 0.92 |  |  |  |  | |  |  |
| NPV= | 0.78 |  |  |  |  | | Previous Sample | |
| Sensitivity= | 0.43 |  |  |  |  | | Error | No Error |
| Specificity= | 0.98 |  |  | Reviewer Using Safer Dx Instrument | Error | | 55 | 5 |
| Overall Accuracy= | 0.80 |  |  |  | No Error | | 74 | 255 |
|  |  |  |  |  |  | |  |  |
| Assuming 1 Error; 2-6 No Error (Most Conservative Interpretation of Error) | | | | | | | |  |
| PPV= | 0.93 |  |  |  |  | |  |  |
| NPV= | 0.75 |  |  |  |  | | Previous Sample | |
| Sensitivity= | 0.33 |  |  |  |  | | Error | No Error |
| Specificity= | 0.99 |  |  | Reviewer Using Safer Dx Instrument | Error | | 42 | 3 |
| Overall Accuracy= | 0.77 |  |  |  | No Error | | 87 | 257 |
|  |  |  |  |  | |  |  |  |

Reference List

(1) Singh H, Giardina TD, Forjuoh SN et al. Electronic health record-based surveillance of diagnostic errors in primary care. *BMJ Qual Saf* 2012;21:93-100.

(2) Singh H, Giardina TD, Meyer AN, Forjuoh SN, Reis MD, Thomas EJ. Types and origins of diagnostic errors in primary care settings. *JAMA Intern Med* 2013;173:418-425.

(3) Singh H, Thomas EJ, Khan MM, Petersen LA. Identifying diagnostic errors in primary care using an electronic screening algorithm. *Arch Intern Med* 2007;167:302-308.
